# Crystal structure of 3-benzyl-2-[(*E*)-2-(furan-2-yl)ethen­yl]-2,3-di­hydro­quinazolin-4(1*H*)-one and 3-benzyl-2-[(*E*)-2-(thio­phen-2-yl)ethen­yl]-2,3-di­hydro­quinazolin-4(1*H*)-one from synchrotron X-ray diffraction

**DOI:** 10.1107/S2056989017017479

**Published:** 2018-01-01

**Authors:** Flavien A. A. Toze, Vladimir P. Zaytsev, Lala V. Chervyakova, Elisaveta A. Kvyatkovskaya, Pavel V. Dorovatovskii, Victor N. Khrustalev

**Affiliations:** a Department of Chemistry, Faculty of Sciences, University of Douala, PO Box 24157, Douala, Republic of Cameroon; b Organic Chemistry Department, Peoples’ Friendship University of Russia (RUDN University), 6 Miklukho-Maklay St., Moscow 117198, Russian Federation; c National Research Centre "Kurchatov Institute", 1 Acad. Kurchatov Sq., Moscow 123182, Russian Federation; d Inorganic Chemistry Department, Peoples’ Friendship University of Russia (RUDN University), 6 Miklukho-Maklay St., Moscow 117198, Russian Federation

**Keywords:** crystal structure, 2-ethenylquinazolines, furyl-acrolein, thienyl-acrolein, three-component reaction, synchrotron radiation

## Abstract

The chiral title compounds, C_21_H_18_N_2_O_2_, (I), and C_21_H_18_N_2_OS, (II) – products of the three-component reaction between benzyl­amine, isatoic anhydride and furyl- or thienyl-acrolein – are isostructural and form isomorphous racemic crystals. The tetra­hydro­pyrimidine ring in (I) and (II) adopts a sofa conformation. The amino N atom has a trigonal–pyramidal geometry [sum of the bond angles is 347.0° for both (I) and (II)], whereas the amido N atom is flat [sum of the bond angles is 359.3° for both (I) and (II)]. The furyl- and thienylethenyl substituents in (I) and (II) are planar and the conformation about the bridging C=C bond is *E*. These bulky fragments occupy the axial position at the quaternary C atom of the tetra­hydro­pyrimidine ring, apparently, due to steric reasons. In the crystals, mol­ecules of (I) and (II) form hydrogen-bonded helicoidal chains propagating along [010] by strong inter­molecular N—H⋯O hydrogen bonds.

## Abstract 2

The mol­ecular and crystal structures of two 3-benzyl-2-[(*E*)-2-(2-ar­yl)ethen­yl]-2,3-di­hydro­quinazolin-4-ones – products of three-component reactions between benzyl­amine, isatoic anhydride and furyl- or thienyl-acrolein in the presence of catalytic qu­antity of *p*-TsOH – were studied by X-ray diffraction.

## Chemical context   

The synthesis and chemistry of quinazoline and quinazolinone derivatives have remained at the focus of biochemical research over the past decade owing to their high and diverse physiological activities (for recent reviews, see: Jafari *et al.*, 2016 ▸; Wang & Gao, 2013 ▸; Selvam & Kumar, 2011 ▸). A large part of these studies has been aimed at the development of methods for the synthesis of 2-aryl-substituted quinazolines. However, 2-ethenylquinazolines are much more attractive synthons for subsequent modifications of the heterocyclic skeleton.

**Figure 1:**
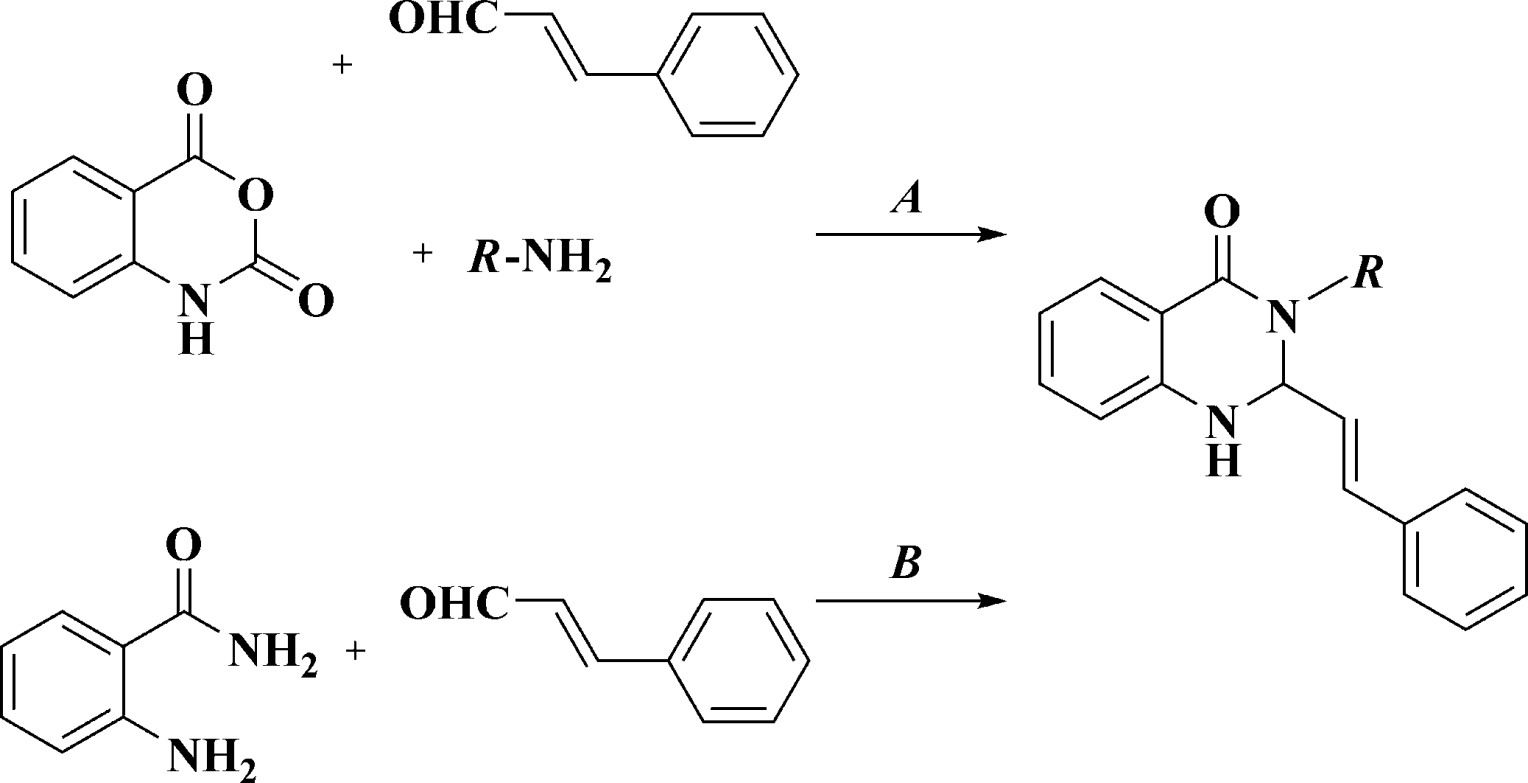
The two general methods, *A* and *B*, for the synthesis of 3-benzyl-2-[(*E*)-2-(2-ar­yl)ethen­yl]-2,3-di­hydro­quinazolin-4(1*H*)-ones (I) and (II).

Two synthetic approaches *A* and *B* (Fig. 1 ▸) are known for 2-ethenylphenyl-substituted heterocycles (Mohammadpoor-Baltork *et al.*, 2011 ▸; Ramesh *et al.*, 2012 ▸; Cheng *et al.*, 2012 ▸; Ghorbani-Choghamarani & Norouzi, 2014 ▸; Zhang *et al.*, 2014 ▸, 2016 ▸; Deng *et al.*, 2015 ▸; Noori *et al.*, 2017 ▸; Alinezhad *et al.*, 2017 ▸). However, up to date, there is practically no information about the synthesis of 2-ethenylhetaryl-substituted quinazolines (Frackenpohl *et al.*, 2016 ▸; Zaytsev *et al.*, 2015 ▸; Celltech & Limited, 2004 ▸; Kundu & Chaudhuri, 2001 ▸). Taking into account the high biological activity of furan, thio­phene, and pyrrole derivatives, it appeared very attractive to obtain quinazolines of this type. It is well known that, for biological researches, the conformation of a mol­ecule plays a key role. In this connection, the present work is aimed at revealing the conformational features of 2-ethenylhetaryl-substituted quinazolines.

Using method *A*, the three-component reaction between benzyl­amine, isatoic anhydride and furyl- or thienylacrolein in the presence of a catalytic qu­antity of *p*-TsOH afforded the 3-benzyl-2-[(*E*)-2-(furan-2-yl)ethen­yl]-2,3-di­hydro­quinazolin-4(1*H*)-one (I) and 3-benzyl-2-[(*E*)-2-(thio­phen-2-yl)ethen­yl]-2,3-di­hydro­quinazolin-4(1*H*)-one (II) in moderate yields.
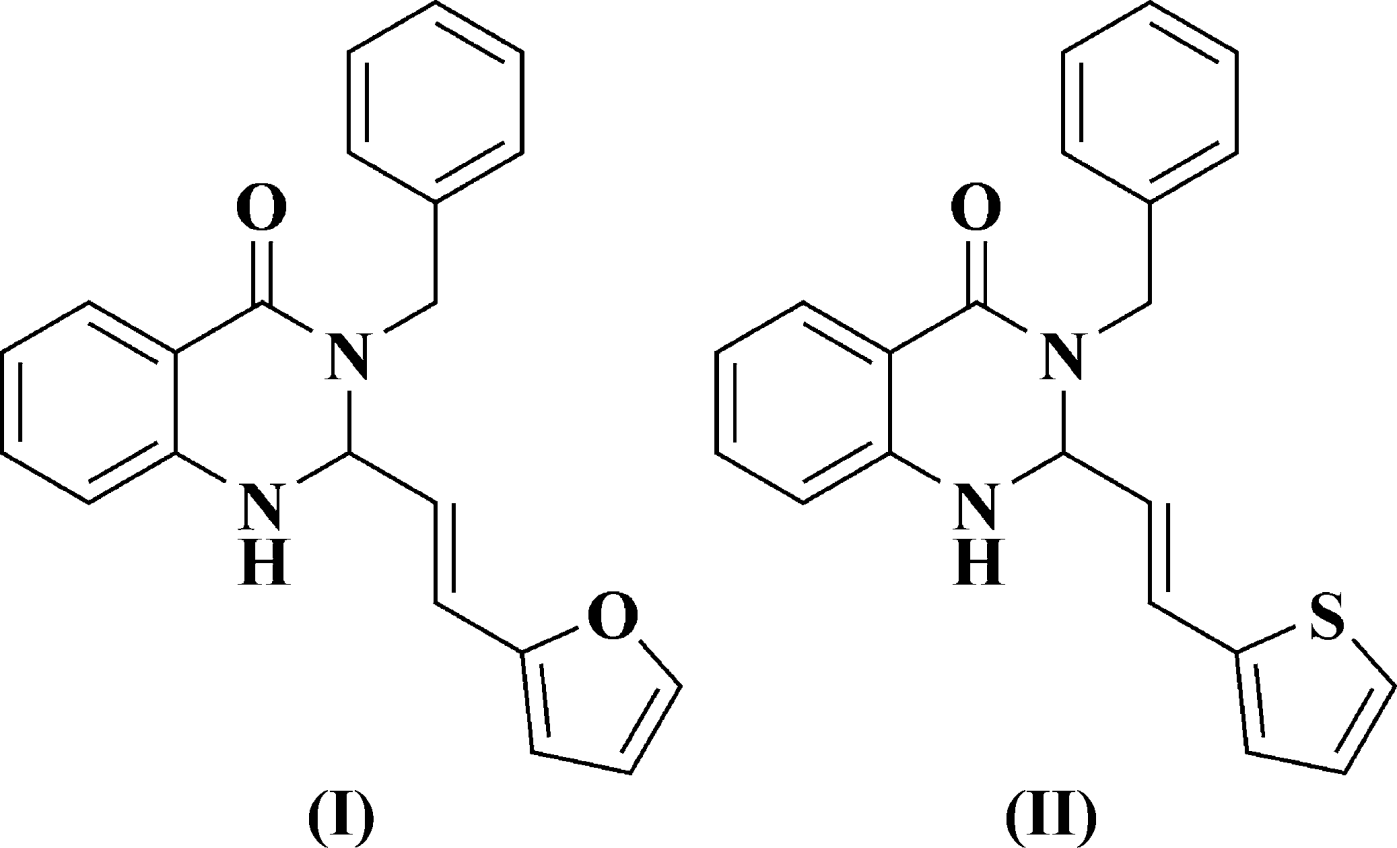


## Structural commentary   

**Figure 2:**
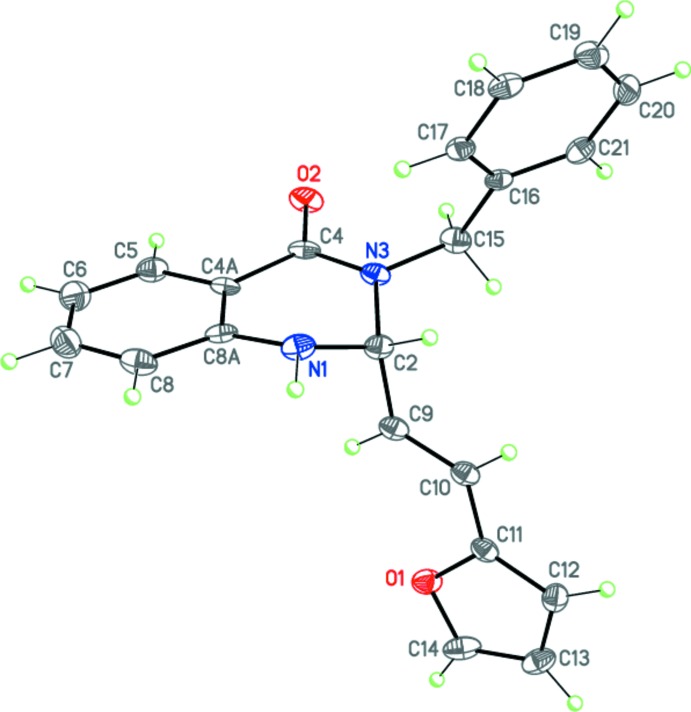
The mol­ecular structure of (I). Displacement ellipsoids are shown at the 50% probability level. H atoms are presented as small spheres of arbitrary radius.

Compounds (I), C_21_H_18_N_2_O_2_, and (II), C_21_H_18_N_2_OS – the products of the three-component reaction between benzyl­amine, isatoic anhydride and furyl- or thienyl-acrolein are isostructural and crystallize in the ortho­rhom­bic space group *Pbca* (Figs. 2 ▸ and 3 ▸).

The tetra­hydro­pyrimidine ring in (I) and (II) adopts a *sofa* conformation, with the C2 carbon atom deviating from the mean plane of the other atoms of the ring by 0.526 (1) and 0.528 (2) Å for (I) and (II), respectively. The nitro­gen N1 atom has a trigonal-pyramidal geometry [sum of the bond angles is 347° for both (I) and (II)], whereas the nitro­gen N3 atom is flattened [sum of the bond angles is 359.3° for both (I) and (II)]. The furyl- and thienyl-ethenyl substituents in (I) and (II) are planar and have the *E*-conformation at the C9=C10 double bond. Remarkably, these bulky fragments occupy the axial position at the quaternary C2 carbon atom of the tetra­hydro­pyrimidine ring, apparently, due to the steric inter­action with the benzyl substituent.

The mol­ecules of (I) and (II) possess an asymmetric center at the C2 carbon atom. The crystals of (I) and (II) are racemates.

## Supra­molecular features   

**Figure 4:**
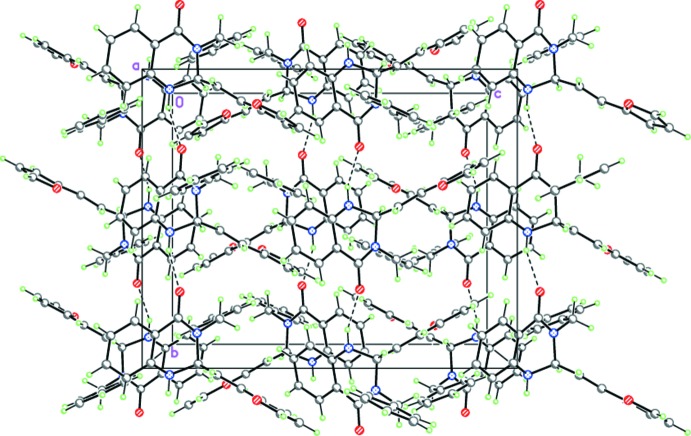
The crystal structure of (I), demonstrating the hydrogen-bonded helicoidal chains propagating in the [010] direction. Dashed lines indicate the inter­molecular N—H⋯O hydrogen bonds.

In the crystals of (I) and (II), mol­ecules form hydrogen-bonded helicoidal chains propagating along the [010] direction by strong inter­molecular N—H⋯O hydrogen bonds (Tables 1 ▸ and 2 ▸, Figs. 4 ▸ and 5 ▸).

## Synthesis and crystallization   

**Figure 6:**
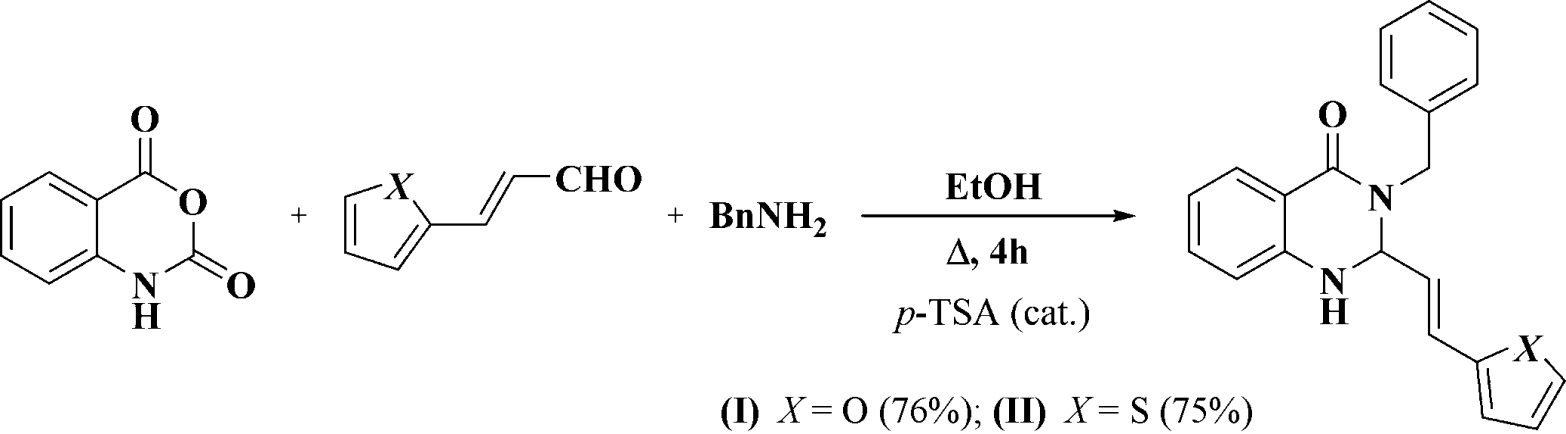
Syntheses of 3-benzyl-2-[(*E*)-2-(furan-2-yl)ethen­yl]-2,3-di­hydro­quin­az­o­lin-4(1*H*)-one (I) and 3-benzyl-2-[(*E*)-2-(thio­phen-2-yl)ethen­yl]-2,3-di­hydro­quinazolin-4(1*H*)-one (II).

3-Benzyl-2-[(*E*)-2-(2-ar­yl)ethen­yl]-2,3-di­hydro­quinazolin-4-ones (I) and (II) were synthesized using a method similar to the recently described procedure (Fig. 6 ▸) (Zaytsev *et al.*, 2017 ▸).

**General procedure.** *p*-TsOH (0.79 g, 4.6 mmol) was added to a mixture of isatoic anhydride (1.5 g, 9.2 mmol), benzyl­amine (1.2 mL, 11.0 mmol), and furyl- or thienylacrolein (9.2 mmol) in 50 mL EtOH. The reaction mixture was heated under reflux for 4 h. The progress of the reaction was monitored by TLC. When the reaction completed, the mixture was diluted with H_2_O (100 mL) and extracted with EtOAc (3 × 50 mL). The organic layers were combined, dried (MgSO_4_), concentrated *in vacuo* and the residue was purified by column chromatography on SiO_2_ (3 × 20 cm) using hexane and then EtOAc/hexane (1/10→1/5) mixtures as eluent. The resulting product was recrystallized from a mixture of hexa­ne–EtOAc [for (I)] or EtOAc–EtOH [for (II)] to afford the analytically pure samples of the target products.

**3-Benzyl-2-[(** ***E*** **)-2-(furan-2-yl)ethen­yl]-2,3-di­hydro­quin­az­olin-4(1** ***H*** **)-one (I)**. Colourless prisms. Yield is 2.31 g (76%). M.p. = 427.1 K (hexa­ne–EtOAc). IR (KBr), ν (cm^−1^): 3376, 1645, 1611. ^1^H NMR (CDCl_3_, 600.2 MHz, 301 K): δ = 3.86 (*d*, 1H, CH_2_N, *J* = 15.1), 4.61 (*br s*, 1H, NH), 4.98 (*br d*, 1H, H2, *J* = 5.5), 5.59 (*d*, 1H, CH_2_N, *J* = 15.1), 6.24 (*d*, 1H, H3, furyl, *J* = 3.1), 6.25 (*d*, 1H, CH=CH, *J* = 6.2), 6.34 (*dd*, 1H, H4, furyl, *J* = 2.1, *J* = 3.1), 6.59 (*d*, 1H, H8, *J* = 8.2), 6.83 (*t*, 1H, H6, *J* = 7.6), 7.24–7.34 (*m*, 7H, HAr), 7.96 (*dd*, 1H, H5, *J* = 1.4, *J* = 7.6). ^13^C NMR (CDCl_3_, 100 MHz, 301 K): δ = 46.7 (CH_2_N), 69.8 (C2), 109.9, 111.5, 114.8, 119.1, 121.1, 123.6, 127.5, 127.9, 128.7, 128.7, 133.6, 115.7, 136.9, 145.4, 151.1, 142.7 (CAr, CH=CH), 162.9 (NCO). MS (EI, 70 eV): *m*/*z* = 330 [*M*]^+^ (93), 239 (100), 197 (71), 170 (20), 160 (19), 120 (40), 106 (55), 91 (81), 76 (58), 65 (45), 51 (37), 43 (20).

**3-Benzyl-2-[(** ***E*** **)-2-(thio­phen-2-yl)ethen­yl]-2,3-di­hydro­quinazolin-4(1** ***H*** **)-one (II)**. Yellow prisms. Yield is 2.39 g (75%). M.p. = 434.1–435.1 K (EtOAc–EtOH). IR (KBr), ν (cm^−1^): 3306, 1625, 1506. ^1^H NMR (DMSO, 600.2 MHz, 301 K): δ = 4.05 (*d*, 1H, CH_2_N, *J* = 15.8), 5.15-5.17 (*m*, 2H, H2, CH_2_N), 6.00 (*dd*, 1H, CH=CH, *J* = 6.8, *J* = 15.1), 6.69–6.76 (*m*, 3H, H6, H8, CH=CH), 6.96 (*dd*, 1H, H4, thienyl, *J* = 3.4, *J* = 5.2), 7.07 (*br d*, 1H, H3, thienyl, *J* = 3.4), 7.07 (*br s*, 1H, NH), 7.23–7.32 (*m*, 6H, HAr), 7.38 (*br d*, 1H, H2, thienyl, *J* = 5.2), 7.66 (*dd*, 1H, H5, *J* = 1.4, *J* = 8.2). ^13^C NMR (DMSO, 150.9 MHz, 301 K): δ = 47.0 (CH_2_N), 69.6 (C2), 115.1, 115.2, 118.0, 125.7 (2C), 126.4, 127.7, 127.9, 128.1, 128.3, 128.4, 129.0, 134.0, 138.3, 140.8, 147.1 (CAr, CH=CH), 162.4 (NCO). MS (EI, 70 eV): *m*/*z* = 346 [*M*]^+^ (76), 255 (100), 237 (93), 213 (37), 106 (14), 91 (99), 65 (13).

## Refinement   

Crystal data, data collection and structure refinement details are summarized in Table 3 ▸. X-ray diffraction studies were carried out on the "Belok" beamline of the National Research Center "Kurchatov Institute" (Moscow, Russian Federation) using a Rayonix SX165 CCD detector. A total of 360 images for each compounds were collected using an oscillation range of 1.0° (φ scan mode, two different crystal orientations) and corrected for absorption using the *SCALA* program (Evans, 2006 ▸). The data were indexed, integrated and scaled using the utility *i*MOSFLM in the *CCP4* program (Battye *et al.*, 2011 ▸).

The hydrogen atoms of the amino groups were localized in difference-Fourier maps and refined isotropically with fixed displacement parameters [*U*_ĩso_(H) = 1.2*U*_eq_(N)]. The other hydrogen atoms were placed in calculated positions with C—H = 0.95–1.00 Å and refined in the riding model with fixed isotropic displacement parameters [*U*_ĩso_(H) = 1.2*U*_eq_(C)].

A relatively large number of reflections (a few dozen) were omitted due to the following reasons: (1) In order to achieve better *I*/σ statistics for high-angle reflections we selected a larger exposure time, which resulted in some intensity overloads in the low-angle part of the area. These corrupted intensities were excluded from final steps of the refinement. (2) In the current setup of the instrument, the low-temperature device eclipses a small region of the detector near its high-angle limit. This resulted in zero intensity of some reflections. (3) In the case of (II), the quality of the single crystal chosen for the diffraction experiment was far from perfect. Some systematic intensity deviations can be due to extinction and defects present in the crystal.

## Supplementary Material

Crystal structure: contains datablock(s) global, I, II. DOI: 10.1107/S2056989017017479/ld2142sup1.cif

Structure factors: contains datablock(s) I. DOI: 10.1107/S2056989017017479/ld2142Isup2.hkl

Structure factors: contains datablock(s) II. DOI: 10.1107/S2056989017017479/ld2142IIsup3.hkl

Supporting information file. DOI: 10.1107/S2056989017017479/ld2142Isup4.cml

Supporting information file. DOI: 10.1107/S2056989017017479/ld2142IIsup5.cml

CCDC references: 1589395, 1589394

Additional supporting information:  crystallographic information; 3D view; checkCIF report

## Figures and Tables

**Figure 3:**
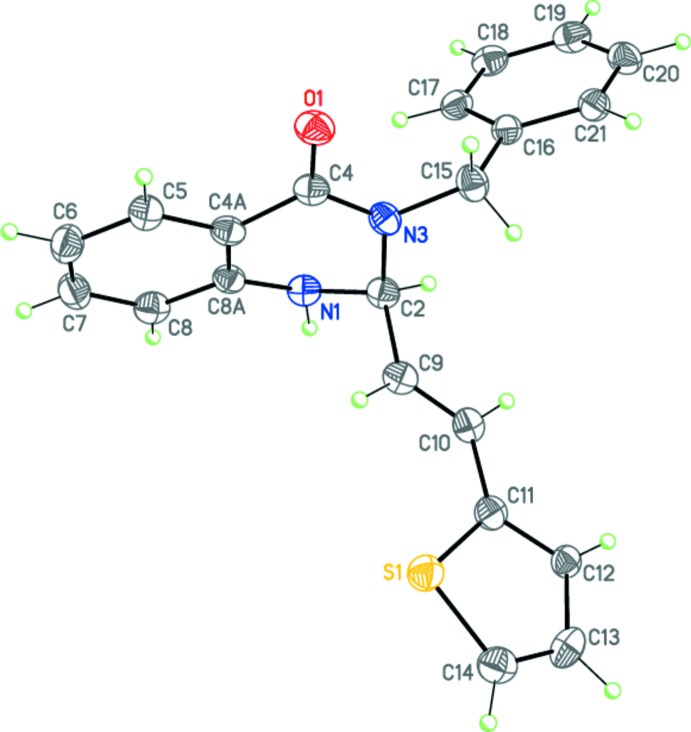
The mol­ecular structure of (II). Displacement ellipsoids are shown at the 50% probability level. H atoms are presented as small spheres of arbitrary radius.

**Figure 5:**
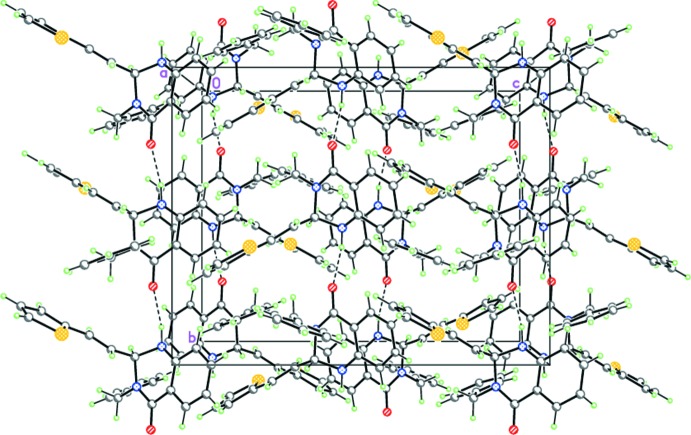
The crystal structure of (II), demonstrating the hydrogen-bonded helicoidal chains propagating in the [010] direction. Dashed lines indicate the inter­molecular N—H⋯O hydrogen bonds.

**Table 1:** Hydrogen-bond geometry (Å, °) for (I)

*D*—H⋯*A*	*D*—H	H⋯*A*	*D*⋯*A*	*D*—H⋯*A*
N1—H1⋯O2^i^	0.897 (15)	2.111 (15)	2.9557 (14)	156.7 (12)

Symmetry code: (i) 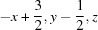.

**Table 2:** Hydrogen-bond geometry (Å, °) for (II)

*D*—H⋯*A*	*D*—H	H⋯*A*	*D*⋯*A*	*D*—H⋯*A*
N1—H1⋯O1^i^	0.87 (3)	2.14 (3)	2.978 (2)	161 (2)

Symmetry code: (i) 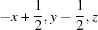.

**Table 3:** Experimental details

	(I)	(II)
Crystal data		
Chemical formula	C_21_H_18_N_2_O_2_	C_21_H_18_N_2_OS
*M*_r_	330.37	346.43
Crystal system, space group	Orthorhombic, *P* *b* *c* *a*	Orthorhombic, *P* *b* *c* *a*
Temperature (K)	100	100
*a*, *b*, *c* (Å)	14.292 (3), 13.729 (3), 17.230 (3)	14.245 (3), 13.855 (3), 17.629 (4)
*V* (Å^3^)	3380.8 (12)	3479.3 (13)
*Z*	8	8
Radiation type	Synchrotron, λ = 0.96260 Å	Synchrotron, λ = 0.96260 Å
μ (mm^−1^)	0.17	0.44
Crystal size (mm)	0.30 × 0.25 × 0.15	0.30 × 0.25 × 0.25

Data collection		
Diffractometer	Rayonix SX165 CCD	Rayonix SX165 CCD
Absorption correction	Multi-scan (*SCALA*; Evans, 2006 ▸)	Multi-scan (*SCALA*; Evans, 2006 ▸)
*T*_min_, *T*_max_	0.940, 0.970	0.870, 0.890
No. of measured, independent and observed [*I* > 2σ(*I*)] reflections	34783, 3705, 3017	20322, 3594, 3024
*R*_int_	0.079	0.064
(sin θ/λ)_max_ (Å^−1^)	0.646	0.647

Refinement		
*R*[*F* ^2^ > 2σ(*F* ^2^)], *wR*(*F* ^2^), *S*	0.042, 0.112, 1.08	0.050, 0.147, 1.08
No. of reflections	3705	3594
No. of parameters	230	217
H-atom treatment	H atoms treated by a mixture of independent and constrained refinement	H atoms treated by a mixture of independent and constrained refinement
Δρ_max_, Δρ_min_ (e Å^−3^)	0.28, −0.17	0.71, −0.72

Computer programs: *Marccd* (Doyle, 2011 ▸), *i* *MOSFLM* (Battye *et al.*, 2011 ▸), *SHELXT* (Sheldrick, 2015 ▸), *SHELXL2014* (Sheldrick, 2015 ▸) and *SHELXTL* (Sheldrick, 2015 ▸).

## supplementary crystallographic information

### 3-Benzyl-2-[(E)-2-(furan-2-yl)ethenyl]-2,3-dihydroquinazolin-4(1H)-one (I) Crystal data

**Table:**

C_21_H_18_N_2_O_2_	*D*_x_ = 1.298 Mg m^−^^3^
*M**_r_* = 330.37	Synchrotron radiation, λ = 0.96260 Å
Orthorhombic, *P**b**c**a*	Cell parameters from 600 reflections
*a* = 14.292 (3) Å	θ = 3.0–36.0°
*b* = 13.729 (3) Å	µ = 0.17 mm^−^^1^
*c* = 17.230 (3) Å	*T* = 100 K
*V* = 3380.8 (12) Å^3^	Prism, colourless
*Z* = 8	0.30 × 0.25 × 0.15 mm
*F*(000) = 1392	

### 3-Benzyl-2-[(E)-2-(furan-2-yl)ethenyl]-2,3-dihydroquinazolin-4(1H)-one (I) Data collection

**Table:**

Rayonix SX165 CCD diffractometer	3017 reflections with *I* > 2σ(*I*)
/f scan	*R*_int_ = 0.079
Absorption correction: multi-scan (Scala; Evans, 2006)	θ_max_ = 38.5°, θ_min_ = 3.2°
*T*_min_ = 0.940, *T*_max_ = 0.970	*h* = −18→18
34783 measured reflections	*k* = −17→17
3705 independent reflections	*l* = −21→21

### 3-Benzyl-2-[(E)-2-(furan-2-yl)ethenyl]-2,3-dihydroquinazolin-4(1H)-one (I) Refinement

**Table:**

Refinement on *F*^2^	Secondary atom site location: difference Fourier map
Least-squares matrix: full	Hydrogen site location: mixed
*R*[*F*^2^ > 2σ(*F*^2^)] = 0.042	H atoms treated by a mixture of independent and constrained refinement
*wR*(*F*^2^) = 0.112	*w* = 1/[σ^2^(*F*_o_^2^) + (0.0539*P*)^2^ + 0.539*P*] where *P* = (*F*_o_^2^ + 2*F*_c_^2^)/3
*S* = 1.08	(Δ/σ)_max_ = 0.001
3705 reflections	Δρ_max_ = 0.28 e Å^−^^3^
230 parameters	Δρ_min_ = −0.17 e Å^−^^3^
0 restraints	Extinction correction: SHELXL2014 (Sheldrick, 2015), Fc^*^=kFc[1+0.001xFc^2^λ^3^/sin(2θ)]^-1/4^
Primary atom site location: difference Fourier map	Extinction coefficient: 0.0041 (8)

### 3-Benzyl-2-[(E)-2-(furan-2-yl)ethenyl]-2,3-dihydroquinazolin-4(1H)-one (I) Special details

**Table:**

Geometry. All esds (except the esd in the dihedral angle between two l.s. planes) are estimated using the full covariance matrix. The cell esds are taken into account individually in the estimation of esds in distances, angles and torsion angles; correlations between esds in cell parameters are only used when they are defined by crystal symmetry. An approximate (isotropic) treatment of cell esds is used for estimating esds involving l.s. planes.

### 3-Benzyl-2-[(E)-2-(furan-2-yl)ethenyl]-2,3-dihydroquinazolin-4(1H)-one (I) Fractional atomic coordinates and isotropic or equivalent isotropic displacement parameters (Å^2^)

**Table:**

	*x*	*y*	*z*	*U*_iso_*/*U*_eq_	
O1	0.94874 (5)	0.38755 (6)	0.80431 (5)	0.0290 (2)	
O2	0.78370 (6)	0.74574 (6)	0.58408 (5)	0.0324 (2)	
N1	0.75445 (7)	0.45360 (8)	0.55253 (6)	0.0259 (3)	
H1	0.7542 (10)	0.3883 (11)	0.5507 (8)	0.031*	
C2	0.73864 (8)	0.49053 (9)	0.63117 (7)	0.0245 (3)	
H2	0.6772	0.4641	0.6493	0.029*	
N3	0.72977 (6)	0.59857 (7)	0.62784 (6)	0.0241 (2)	
C4	0.78346 (8)	0.65486 (9)	0.57961 (7)	0.0243 (3)	
C4A	0.83803 (7)	0.60140 (8)	0.51944 (7)	0.0239 (3)	
C5	0.89993 (8)	0.65246 (9)	0.47069 (8)	0.0287 (3)	
H5	0.9115	0.7196	0.4800	0.034*	
C6	0.94453 (8)	0.60605 (10)	0.40899 (8)	0.0336 (3)	
H6	0.9863	0.6409	0.3763	0.040*	
C7	0.92651 (9)	0.50687 (10)	0.39610 (8)	0.0343 (3)	
H7	0.9564	0.4747	0.3541	0.041*	
C8	0.86562 (8)	0.45466 (9)	0.44362 (7)	0.0296 (3)	
H8	0.8545	0.3875	0.4338	0.036*	
C8A	0.82023 (7)	0.50135 (9)	0.50644 (7)	0.0240 (3)	
C9	0.81224 (8)	0.45883 (8)	0.68936 (7)	0.0243 (3)	
H9	0.8760	0.4743	0.6798	0.029*	
C10	0.78969 (8)	0.40943 (9)	0.75408 (7)	0.0253 (3)	
H10	0.7251	0.3963	0.7618	0.030*	
C11	0.85346 (8)	0.37393 (9)	0.81353 (7)	0.0252 (3)	
C12	0.83634 (9)	0.32594 (9)	0.88182 (7)	0.0295 (3)	
H12	0.7769	0.3077	0.9017	0.035*	
C13	0.92549 (9)	0.30860 (9)	0.91761 (8)	0.0328 (3)	
H13	0.9367	0.2768	0.9657	0.039*	
C14	0.99049 (9)	0.34671 (9)	0.86917 (8)	0.0321 (3)	
H14	1.0560	0.3456	0.8784	0.039*	
C15	0.67156 (8)	0.64346 (9)	0.68802 (7)	0.0271 (3)	
H15A	0.6881	0.7133	0.6921	0.032*	
H15B	0.6860	0.6125	0.7385	0.032*	
C16	0.56621 (8)	0.63468 (8)	0.67274 (7)	0.0232 (3)	
C17	0.52921 (8)	0.59729 (9)	0.60354 (7)	0.0255 (3)	
H17	0.5702	0.5750	0.5639	0.031*	
C18	0.43186 (8)	0.59258 (9)	0.59243 (8)	0.0287 (3)	
H18	0.4074	0.5664	0.5456	0.034*	
C19	0.37128 (8)	0.62615 (9)	0.64965 (8)	0.0319 (3)	
H19	0.3055	0.6233	0.6418	0.038*	
C20	0.40750 (9)	0.66416 (9)	0.71896 (8)	0.0316 (3)	
H20	0.3663	0.6876	0.7580	0.038*	
C21	0.50438 (8)	0.66754 (9)	0.73049 (7)	0.0272 (3)	
H21	0.5286	0.6923	0.7779	0.033*	

### 3-Benzyl-2-[(E)-2-(furan-2-yl)ethenyl]-2,3-dihydroquinazolin-4(1H)-one (I) Atomic displacement parameters (Å^2^)

**Table:**

	*U*^11^	*U*^22^	*U*^33^	*U*^12^	*U*^13^	*U*^23^
O1	0.0247 (4)	0.0273 (5)	0.0350 (5)	−0.0009 (3)	−0.0026 (4)	0.0020 (4)
O2	0.0333 (5)	0.0183 (5)	0.0457 (6)	0.0014 (3)	0.0002 (4)	−0.0013 (4)
N1	0.0288 (5)	0.0170 (5)	0.0319 (6)	−0.0020 (4)	−0.0042 (4)	−0.0008 (4)
C2	0.0222 (5)	0.0201 (6)	0.0311 (7)	−0.0013 (4)	−0.0017 (5)	0.0008 (5)
N3	0.0208 (5)	0.0193 (5)	0.0322 (6)	0.0012 (4)	−0.0011 (4)	−0.0016 (4)
C4	0.0208 (5)	0.0188 (6)	0.0335 (7)	0.0010 (4)	−0.0060 (5)	0.0002 (5)
C4A	0.0200 (5)	0.0213 (6)	0.0303 (7)	0.0016 (4)	−0.0045 (5)	0.0009 (5)
C5	0.0231 (5)	0.0247 (7)	0.0385 (7)	0.0003 (5)	−0.0042 (5)	0.0036 (5)
C6	0.0256 (6)	0.0383 (8)	0.0370 (8)	0.0006 (5)	0.0015 (5)	0.0044 (6)
C7	0.0283 (6)	0.0421 (8)	0.0326 (7)	0.0059 (6)	−0.0002 (5)	−0.0057 (6)
C8	0.0274 (6)	0.0263 (7)	0.0351 (7)	0.0033 (5)	−0.0062 (5)	−0.0055 (5)
C8A	0.0215 (5)	0.0219 (6)	0.0285 (7)	0.0018 (4)	−0.0075 (5)	0.0007 (5)
C9	0.0214 (5)	0.0212 (6)	0.0305 (7)	−0.0006 (4)	−0.0013 (5)	−0.0027 (5)
C10	0.0224 (5)	0.0225 (6)	0.0311 (7)	0.0001 (4)	−0.0002 (5)	−0.0031 (5)
C11	0.0248 (5)	0.0220 (6)	0.0289 (7)	−0.0002 (5)	0.0008 (5)	−0.0042 (5)
C12	0.0326 (6)	0.0285 (7)	0.0274 (7)	−0.0013 (5)	0.0018 (5)	−0.0019 (5)
C13	0.0430 (7)	0.0279 (7)	0.0276 (7)	−0.0010 (6)	−0.0090 (6)	−0.0003 (5)
C14	0.0308 (6)	0.0262 (7)	0.0394 (8)	0.0004 (5)	−0.0122 (6)	0.0010 (6)
C15	0.0234 (6)	0.0266 (7)	0.0312 (7)	0.0010 (5)	−0.0025 (5)	−0.0047 (5)
C16	0.0232 (5)	0.0192 (6)	0.0273 (6)	0.0000 (4)	−0.0018 (5)	0.0032 (5)
C17	0.0263 (6)	0.0234 (6)	0.0268 (7)	0.0019 (5)	−0.0015 (5)	0.0021 (5)
C18	0.0287 (6)	0.0253 (7)	0.0320 (7)	−0.0016 (5)	−0.0070 (5)	0.0049 (5)
C19	0.0200 (5)	0.0332 (7)	0.0426 (8)	−0.0018 (5)	−0.0019 (5)	0.0100 (6)
C20	0.0275 (6)	0.0312 (7)	0.0360 (8)	0.0004 (5)	0.0090 (5)	0.0063 (6)
C21	0.0296 (6)	0.0246 (7)	0.0274 (7)	−0.0016 (5)	0.0018 (5)	0.0033 (5)

### 3-Benzyl-2-[(E)-2-(furan-2-yl)ethenyl]-2,3-dihydroquinazolin-4(1H)-one (I) Geometric parameters (Å, º)

**Table:**

O1—C11	1.3836 (14)	C9—H9	0.9500
O1—C14	1.3854 (16)	C10—C11	1.4551 (17)
O2—C4	1.2501 (15)	C10—H10	0.9500
N1—C8A	1.3943 (16)	C11—C12	1.3707 (18)
N1—C2	1.4643 (17)	C12—C13	1.4354 (18)
N1—H1	0.897 (15)	C12—H12	0.9500
C2—N3	1.4898 (16)	C13—C14	1.3540 (19)
C2—C9	1.5169 (16)	C13—H13	0.9500
C2—H2	1.0000	C14—H14	0.9500
N3—C4	1.3699 (16)	C15—C16	1.5334 (16)
N3—C15	1.4653 (15)	C15—H15A	0.9900
C4—C4A	1.4905 (17)	C15—H15B	0.9900
C4A—C5	1.4069 (17)	C16—C17	1.4017 (17)
C4A—C8A	1.4149 (17)	C16—C21	1.4051 (17)
C5—C6	1.3938 (19)	C17—C18	1.4058 (16)
C5—H5	0.9500	C17—H17	0.9500
C6—C7	1.403 (2)	C18—C19	1.3907 (18)
C6—H6	0.9500	C18—H18	0.9500
C7—C8	1.3934 (19)	C19—C20	1.4023 (19)
C7—H7	0.9500	C19—H19	0.9500
C8—C8A	1.4154 (17)	C20—C21	1.3996 (17)
C8—H8	0.9500	C20—H20	0.9500
C9—C10	1.3445 (17)	C21—H21	0.9500
			
C11—O1—C14	106.06 (9)	C9—C10—H10	116.5
C8A—N1—C2	117.92 (10)	C11—C10—H10	116.5
C8A—N1—H1	116.9 (9)	C12—C11—O1	109.83 (10)
C2—N1—H1	112.2 (9)	C12—C11—C10	130.80 (11)
N1—C2—N3	108.80 (9)	O1—C11—C10	119.37 (10)
N1—C2—C9	113.91 (9)	C11—C12—C13	106.86 (11)
N3—C2—C9	111.72 (9)	C11—C12—H12	126.6
N1—C2—H2	107.4	C13—C12—H12	126.6
N3—C2—H2	107.4	C14—C13—C12	106.27 (11)
C9—C2—H2	107.4	C14—C13—H13	126.9
C4—N3—C15	120.66 (10)	C12—C13—H13	126.9
C4—N3—C2	122.51 (9)	C13—C14—O1	110.99 (11)
C15—N3—C2	116.09 (10)	C13—C14—H14	124.5
O2—C4—N3	121.82 (11)	O1—C14—H14	124.5
O2—C4—C4A	122.20 (11)	N3—C15—C16	113.75 (10)
N3—C4—C4A	115.92 (10)	N3—C15—H15A	108.8
C5—C4A—C8A	120.15 (11)	C16—C15—H15A	108.8
C5—C4A—C4	119.93 (11)	N3—C15—H15B	108.8
C8A—C4A—C4	119.61 (10)	C16—C15—H15B	108.8
C6—C5—C4A	121.02 (12)	H15A—C15—H15B	107.7
C6—C5—H5	119.5	C17—C16—C21	118.87 (11)
C4A—C5—H5	119.5	C17—C16—C15	123.05 (11)
C5—C6—C7	118.70 (12)	C21—C16—C15	118.07 (11)
C5—C6—H6	120.6	C16—C17—C18	120.40 (11)
C7—C6—H6	120.6	C16—C17—H17	119.8
C8—C7—C6	121.38 (12)	C18—C17—H17	119.8
C8—C7—H7	119.3	C19—C18—C17	120.28 (12)
C6—C7—H7	119.3	C19—C18—H18	119.9
C7—C8—C8A	120.18 (12)	C17—C18—H18	119.9
C7—C8—H8	119.9	C18—C19—C20	119.82 (11)
C8A—C8—H8	119.9	C18—C19—H19	120.1
N1—C8A—C4A	119.18 (11)	C20—C19—H19	120.1
N1—C8A—C8	122.12 (11)	C21—C20—C19	119.90 (12)
C4A—C8A—C8	118.57 (11)	C21—C20—H20	120.1
C10—C9—C2	121.78 (10)	C19—C20—H20	120.1
C10—C9—H9	119.1	C20—C21—C16	120.72 (12)
C2—C9—H9	119.1	C20—C21—H21	119.6
C9—C10—C11	127.06 (11)	C16—C21—H21	119.6
			
C8A—N1—C2—N3	45.76 (13)	C7—C8—C8A—C4A	0.11 (17)
C8A—N1—C2—C9	−79.59 (13)	N1—C2—C9—C10	−122.41 (12)
N1—C2—N3—C4	−38.41 (13)	N3—C2—C9—C10	113.81 (12)
C9—C2—N3—C4	88.20 (13)	C2—C9—C10—C11	179.09 (11)
N1—C2—N3—C15	151.40 (9)	C14—O1—C11—C12	−0.15 (13)
C9—C2—N3—C15	−81.99 (12)	C14—O1—C11—C10	179.98 (10)
C15—N3—C4—O2	−1.24 (16)	C9—C10—C11—C12	177.81 (13)
C2—N3—C4—O2	−171.00 (10)	C9—C10—C11—O1	−2.35 (18)
C15—N3—C4—C4A	−178.63 (9)	O1—C11—C12—C13	0.14 (14)
C2—N3—C4—C4A	11.61 (15)	C10—C11—C12—C13	179.99 (12)
O2—C4—C4A—C5	6.86 (17)	C11—C12—C13—C14	−0.07 (14)
N3—C4—C4A—C5	−175.76 (10)	C12—C13—C14—O1	−0.02 (15)
O2—C4—C4A—C8A	−166.71 (11)	C11—O1—C14—C13	0.10 (14)
N3—C4—C4A—C8A	10.66 (15)	C4—N3—C15—C16	111.21 (12)
C8A—C4A—C5—C6	0.12 (17)	C2—N3—C15—C16	−78.40 (13)
C4—C4A—C5—C6	−173.42 (11)	N3—C15—C16—C17	−6.96 (16)
C4A—C5—C6—C7	0.08 (18)	N3—C15—C16—C21	174.31 (10)
C5—C6—C7—C8	−0.18 (19)	C21—C16—C17—C18	−0.21 (17)
C6—C7—C8—C8A	0.08 (19)	C15—C16—C17—C18	−178.93 (11)
C2—N1—C8A—C4A	−27.67 (15)	C16—C17—C18—C19	0.82 (18)
C2—N1—C8A—C8	156.49 (11)	C17—C18—C19—C20	−0.48 (18)
C5—C4A—C8A—N1	−176.20 (10)	C18—C19—C20—C21	−0.45 (18)
C4—C4A—C8A—N1	−2.63 (15)	C19—C20—C21—C16	1.06 (18)
C5—C4A—C8A—C8	−0.21 (16)	C17—C16—C21—C20	−0.73 (17)
C4—C4A—C8A—C8	173.35 (10)	C15—C16—C21—C20	178.06 (11)
C7—C8—C8A—N1	175.97 (11)		

### 3-Benzyl-2-[(E)-2-(furan-2-yl)ethenyl]-2,3-dihydroquinazolin-4(1H)-one (I) Hydrogen-bond geometry (Å, º)

**Table:**

*D*—H···*A*	*D*—H	H···*A*	*D*···*A*	*D*—H···*A*
N1—H1···O2^i^	0.897 (15)	2.111 (15)	2.9557 (14)	156.7 (12)

Symmetry code: (i) −*x*+3/2, *y*−1/2, *z*.

### 3-Benzyl-2-[(E)-2-(thiophen-2-yl)ethenyl]-2,3-dihydroquinazolin-4(1H)-one (II) Crystal data

**Table:**

C_21_H_18_N_2_OS	*D*_x_ = 1.323 Mg m^−^^3^
*M**_r_* = 346.43	Synchrotron radiation, λ = 0.96260 Å
Orthorhombic, *P**b**c**a*	Cell parameters from 600 reflections
*a* = 14.245 (3) Å	θ = 3.0–33.0°
*b* = 13.855 (3) Å	µ = 0.44 mm^−^^1^
*c* = 17.629 (4) Å	*T* = 100 K
*V* = 3479.3 (13) Å^3^	Prism, yellow
*Z* = 8	0.30 × 0.25 × 0.25 mm
*F*(000) = 1456	

### 3-Benzyl-2-[(E)-2-(thiophen-2-yl)ethenyl]-2,3-dihydroquinazolin-4(1H)-one (II) Data collection

**Table:**

Rayonix SX165 CCD diffractometer	3024 reflections with *I* > 2σ(*I*)
φ scan	*R*_int_ = 0.064
Absorption correction: multi-scan (Scala; Evans, 2006)	θ_max_ = 38.5°, θ_min_ = 3.1°
*T*_min_ = 0.870, *T*_max_ = 0.890	*h* = −15→16
20322 measured reflections	*k* = −17→17
3594 independent reflections	*l* = −15→22

### 3-Benzyl-2-[(E)-2-(thiophen-2-yl)ethenyl]-2,3-dihydroquinazolin-4(1H)-one (II) Refinement

**Table:**

Refinement on *F*^2^	Primary atom site location: difference Fourier map
Least-squares matrix: full	Secondary atom site location: difference Fourier map
*R*[*F*^2^ > 2σ(*F*^2^)] = 0.050	Hydrogen site location: mixed
*wR*(*F*^2^) = 0.147	H atoms treated by a mixture of independent and constrained refinement
*S* = 1.08	*w* = 1/[σ^2^(*F*_o_^2^) + (0.0669*P*)^2^ + 2.4*P*] where *P* = (*F*_o_^2^ + 2*F*_c_^2^)/3
3594 reflections	(Δ/σ)_max_ < 0.001
217 parameters	Δρ_max_ = 0.71 e Å^−^^3^
0 restraints	Δρ_min_ = −0.72 e Å^−^^3^

### 3-Benzyl-2-[(E)-2-(thiophen-2-yl)ethenyl]-2,3-dihydroquinazolin-4(1H)-one (II) Special details

**Table:**

Geometry. All esds (except the esd in the dihedral angle between two l.s. planes) are estimated using the full covariance matrix. The cell esds are taken into account individually in the estimation of esds in distances, angles and torsion angles; correlations between esds in cell parameters are only used when they are defined by crystal symmetry. An approximate (isotropic) treatment of cell esds is used for estimating esds involving l.s. planes.

### 3-Benzyl-2-[(E)-2-(thiophen-2-yl)ethenyl]-2,3-dihydroquinazolin-4(1H)-one (II) Fractional atomic coordinates and isotropic or equivalent isotropic displacement parameters (Å^2^)

**Table:**

	*x*	*y*	*z*	*U*_iso_*/*U*_eq_	
S1	0.02475 (4)	0.39698 (3)	0.79544 (3)	0.02956 (19)	
O1	0.22041 (10)	0.74793 (9)	0.57892 (8)	0.0307 (3)	
N1	0.25131 (12)	0.45803 (11)	0.55151 (9)	0.0249 (4)	
H1	0.2524 (17)	0.3954 (19)	0.5496 (12)	0.030*	
C2	0.26696 (14)	0.49600 (12)	0.62814 (11)	0.0244 (4)	
H2	0.3287	0.4706	0.6463	0.029*	
N3	0.27450 (11)	0.60304 (10)	0.62410 (9)	0.0229 (4)	
C4	0.22059 (13)	0.65770 (13)	0.57606 (10)	0.0231 (4)	
C4A	0.16533 (14)	0.60308 (12)	0.51890 (10)	0.0228 (4)	
C5	0.10052 (15)	0.65191 (14)	0.47233 (11)	0.0276 (4)	
H5	0.0882	0.7184	0.4810	0.033*	
C6	0.05414 (17)	0.60404 (15)	0.41351 (12)	0.0333 (5)	
H6	0.0103	0.6371	0.3823	0.040*	
C7	0.07389 (16)	0.50578 (16)	0.40152 (12)	0.0336 (5)	
H7	0.0433	0.4727	0.3613	0.040*	
C8	0.13683 (15)	0.45601 (14)	0.44685 (11)	0.0301 (5)	
H8	0.1487	0.3896	0.4376	0.036*	
C8A	0.18367 (13)	0.50393 (12)	0.50701 (10)	0.0234 (4)	
C9	0.19299 (14)	0.46488 (12)	0.68451 (11)	0.0244 (4)	
H9	0.1295	0.4819	0.6749	0.029*	
C10	0.21254 (15)	0.41433 (13)	0.74758 (11)	0.0264 (4)	
H10	0.2767	0.3995	0.7566	0.032*	
C11	0.14428 (15)	0.37959 (13)	0.80424 (10)	0.0256 (4)	
C12	0.16756 (16)	0.32822 (13)	0.87251 (11)	0.0304 (3)	
H12	0.2292	0.3110	0.8879	0.037*	
C13	0.08295 (16)	0.30680 (14)	0.91398 (11)	0.0304 (3)	
H13	0.0830	0.2734	0.9610	0.037*	
C14	0.00234 (17)	0.33875 (14)	0.87974 (11)	0.0304 (3)	
H14	−0.0588	0.3298	0.9002	0.037*	
C15	0.33187 (14)	0.64888 (13)	0.68295 (11)	0.0265 (4)	
H15A	0.3163	0.6195	0.7326	0.032*	
H15B	0.3156	0.7183	0.6856	0.032*	
C16	0.43773 (14)	0.63919 (12)	0.66925 (10)	0.0224 (4)	
C17	0.47531 (15)	0.60700 (13)	0.60001 (11)	0.0261 (4)	
H17	0.4345	0.5901	0.5595	0.031*	
C18	0.57301 (16)	0.59978 (14)	0.59048 (12)	0.0313 (5)	
H18	0.5982	0.5765	0.5441	0.038*	
C19	0.63326 (16)	0.62705 (15)	0.64953 (13)	0.0345 (5)	
H19	0.6993	0.6232	0.6429	0.041*	
C20	0.59602 (16)	0.66002 (14)	0.71824 (13)	0.0330 (5)	
H20	0.6368	0.6793	0.7581	0.040*	
C21	0.49895 (15)	0.66462 (13)	0.72819 (11)	0.0257 (4)	
H21	0.4741	0.6852	0.7755	0.031*	

### 3-Benzyl-2-[(E)-2-(thiophen-2-yl)ethenyl]-2,3-dihydroquinazolin-4(1H)-one (II) Atomic displacement parameters (Å^2^)

**Table:**

	*U*^11^	*U*^22^	*U*^33^	*U*^12^	*U*^13^	*U*^23^
S1	0.0350 (4)	0.0244 (3)	0.0293 (3)	0.00022 (19)	0.0016 (2)	0.00190 (17)
O1	0.0362 (9)	0.0153 (6)	0.0407 (8)	−0.0011 (6)	−0.0010 (6)	−0.0009 (5)
N1	0.0316 (10)	0.0150 (7)	0.0280 (8)	0.0016 (6)	0.0027 (7)	−0.0011 (6)
C2	0.0263 (11)	0.0178 (8)	0.0292 (9)	0.0015 (7)	0.0010 (8)	0.0008 (7)
N3	0.0224 (9)	0.0165 (7)	0.0298 (8)	−0.0006 (6)	−0.0011 (7)	−0.0017 (6)
C4	0.0232 (10)	0.0177 (8)	0.0284 (9)	0.0001 (7)	0.0038 (7)	0.0004 (6)
C4A	0.0245 (11)	0.0186 (8)	0.0253 (9)	−0.0016 (7)	0.0043 (8)	0.0010 (6)
C5	0.0306 (11)	0.0236 (8)	0.0285 (9)	0.0014 (8)	0.0029 (8)	0.0012 (7)
C6	0.0345 (13)	0.0369 (11)	0.0286 (10)	0.0016 (9)	−0.0012 (9)	0.0013 (8)
C7	0.0358 (12)	0.0365 (11)	0.0287 (10)	−0.0053 (9)	−0.0014 (9)	−0.0059 (8)
C8	0.0363 (12)	0.0237 (9)	0.0305 (10)	−0.0043 (8)	0.0063 (8)	−0.0050 (7)
C8A	0.0249 (10)	0.0203 (8)	0.0250 (9)	−0.0031 (7)	0.0058 (7)	0.0005 (7)
C9	0.0237 (10)	0.0201 (8)	0.0295 (9)	0.0012 (7)	0.0022 (8)	−0.0011 (7)
C10	0.0291 (11)	0.0213 (8)	0.0288 (9)	0.0005 (7)	−0.0004 (8)	−0.0020 (7)
C11	0.0325 (12)	0.0194 (8)	0.0248 (9)	0.0009 (8)	−0.0008 (8)	−0.0018 (7)
C12	0.0409 (7)	0.0244 (5)	0.0260 (5)	0.0018 (5)	0.0042 (5)	−0.0015 (4)
C13	0.0409 (7)	0.0244 (5)	0.0260 (5)	0.0018 (5)	0.0042 (5)	−0.0015 (4)
C14	0.0409 (7)	0.0244 (5)	0.0260 (5)	0.0018 (5)	0.0042 (5)	−0.0015 (4)
C15	0.0272 (11)	0.0240 (8)	0.0283 (9)	0.0007 (8)	0.0004 (8)	−0.0044 (7)
C16	0.0239 (10)	0.0173 (8)	0.0260 (9)	−0.0003 (7)	−0.0001 (7)	0.0028 (6)
C17	0.0282 (12)	0.0220 (9)	0.0281 (10)	−0.0006 (7)	0.0025 (8)	0.0037 (7)
C18	0.0339 (12)	0.0258 (9)	0.0341 (10)	0.0020 (8)	0.0087 (9)	0.0066 (7)
C19	0.0259 (11)	0.0284 (9)	0.0492 (12)	0.0014 (8)	0.0031 (10)	0.0107 (9)
C20	0.0327 (13)	0.0254 (9)	0.0409 (11)	−0.0002 (8)	−0.0079 (9)	0.0048 (8)
C21	0.0279 (11)	0.0194 (8)	0.0297 (9)	0.0011 (8)	−0.0022 (8)	0.0021 (7)

### 3-Benzyl-2-[(E)-2-(thiophen-2-yl)ethenyl]-2,3-dihydroquinazolin-4(1H)-one (II) Geometric parameters (Å, º)

**Table:**

S1—C14	1.721 (2)	C9—H9	0.9500
S1—C11	1.727 (2)	C10—C11	1.475 (3)
O1—C4	1.251 (2)	C10—H10	0.9500
N1—C8A	1.396 (3)	C11—C12	1.437 (3)
N1—C2	1.467 (2)	C12—C13	1.440 (3)
N1—H1	0.87 (3)	C12—H12	0.9500
C2—N3	1.489 (2)	C13—C14	1.371 (3)
C2—C9	1.511 (3)	C13—H13	0.9500
C2—H2	1.0000	C14—H14	0.9500
N3—C4	1.371 (2)	C15—C16	1.533 (3)
N3—C15	1.465 (2)	C15—H15A	0.9900
C4—C4A	1.486 (3)	C15—H15B	0.9900
C4A—C5	1.409 (3)	C16—C21	1.402 (3)
C4A—C8A	1.414 (2)	C16—C17	1.406 (3)
C5—C6	1.397 (3)	C17—C18	1.405 (3)
C5—H5	0.9500	C17—H17	0.9500
C6—C7	1.406 (3)	C18—C19	1.401 (3)
C6—H6	0.9500	C18—H18	0.9500
C7—C8	1.385 (3)	C19—C20	1.399 (3)
C7—H7	0.9500	C19—H19	0.9500
C8—C8A	1.418 (3)	C20—C21	1.395 (3)
C8—H8	0.9500	C20—H20	0.9500
C9—C10	1.343 (3)	C21—H21	0.9500
			
C14—S1—C11	92.28 (10)	C9—C10—H10	116.8
C8A—N1—C2	117.34 (15)	C11—C10—H10	116.8
C8A—N1—H1	116.5 (16)	C12—C11—C10	125.22 (19)
C2—N1—H1	113.1 (15)	C12—C11—S1	111.83 (15)
N1—C2—N3	108.92 (14)	C10—C11—S1	122.94 (14)
N1—C2—C9	113.41 (16)	C11—C12—C13	109.51 (19)
N3—C2—C9	111.47 (15)	C11—C12—H12	125.2
N1—C2—H2	107.6	C13—C12—H12	125.2
N3—C2—H2	107.6	C14—C13—C12	114.26 (18)
C9—C2—H2	107.6	C14—C13—H13	122.9
C4—N3—C15	120.68 (15)	C12—C13—H13	122.9
C4—N3—C2	122.63 (15)	C13—C14—S1	112.11 (17)
C15—N3—C2	115.99 (15)	C13—C14—H14	123.9
O1—C4—N3	121.87 (17)	S1—C14—H14	123.9
O1—C4—C4A	122.35 (17)	N3—C15—C16	113.52 (15)
N3—C4—C4A	115.74 (15)	N3—C15—H15A	108.9
C5—C4A—C8A	120.09 (17)	C16—C15—H15A	108.9
C5—C4A—C4	119.86 (16)	N3—C15—H15B	108.9
C8A—C4A—C4	119.83 (17)	C16—C15—H15B	108.9
C6—C5—C4A	120.97 (18)	H15A—C15—H15B	107.7
C6—C5—H5	119.5	C21—C16—C17	119.12 (18)
C4A—C5—H5	119.5	C21—C16—C15	118.25 (17)
C5—C6—C7	118.5 (2)	C17—C16—C15	122.63 (17)
C5—C6—H6	120.8	C18—C17—C16	120.24 (19)
C7—C6—H6	120.8	C18—C17—H17	119.9
C8—C7—C6	121.66 (19)	C16—C17—H17	119.9
C8—C7—H7	119.2	C19—C18—C17	119.90 (19)
C6—C7—H7	119.2	C19—C18—H18	120.0
C7—C8—C8A	120.21 (18)	C17—C18—H18	120.0
C7—C8—H8	119.9	C20—C19—C18	119.9 (2)
C8A—C8—H8	119.9	C20—C19—H19	120.0
N1—C8A—C4A	119.13 (17)	C18—C19—H19	120.0
N1—C8A—C8	122.14 (16)	C21—C20—C19	120.0 (2)
C4A—C8A—C8	118.60 (17)	C21—C20—H20	120.0
C10—C9—C2	123.27 (19)	C19—C20—H20	120.0
C10—C9—H9	118.4	C20—C21—C16	120.79 (19)
C2—C9—H9	118.4	C20—C21—H21	119.6
C9—C10—C11	126.45 (19)	C16—C21—H21	119.6
			
C8A—N1—C2—N3	−46.4 (2)	C7—C8—C8A—C4A	−0.6 (3)
C8A—N1—C2—C9	78.4 (2)	N1—C2—C9—C10	120.5 (2)
N1—C2—N3—C4	37.7 (2)	N3—C2—C9—C10	−116.17 (19)
C9—C2—N3—C4	−88.2 (2)	C2—C9—C10—C11	−178.56 (17)
N1—C2—N3—C15	−151.91 (16)	C9—C10—C11—C12	−177.92 (18)
C9—C2—N3—C15	82.2 (2)	C9—C10—C11—S1	2.0 (3)
C15—N3—C4—O1	2.2 (3)	C14—S1—C11—C12	0.19 (15)
C2—N3—C4—O1	172.24 (17)	C14—S1—C11—C10	−179.70 (16)
C15—N3—C4—C4A	−179.85 (16)	C10—C11—C12—C13	179.56 (17)
C2—N3—C4—C4A	−9.9 (3)	S1—C11—C12—C13	−0.3 (2)
O1—C4—C4A—C5	−8.7 (3)	C11—C12—C13—C14	0.4 (2)
N3—C4—C4A—C5	173.42 (17)	C12—C13—C14—S1	−0.2 (2)
O1—C4—C4A—C8A	165.94 (18)	C11—S1—C14—C13	0.02 (16)
N3—C4—C4A—C8A	−11.9 (3)	C4—N3—C15—C16	−112.23 (18)
C8A—C4A—C5—C6	−0.6 (3)	C2—N3—C15—C16	77.1 (2)
C4—C4A—C5—C6	174.00 (18)	N3—C15—C16—C21	−168.65 (15)
C4A—C5—C6—C7	−0.3 (3)	N3—C15—C16—C17	12.1 (2)
C5—C6—C7—C8	0.7 (3)	C21—C16—C17—C18	0.5 (3)
C6—C7—C8—C8A	−0.3 (3)	C15—C16—C17—C18	179.79 (16)
C2—N1—C8A—C4A	28.7 (2)	C16—C17—C18—C19	−1.6 (3)
C2—N1—C8A—C8	−155.42 (18)	C17—C18—C19—C20	1.0 (3)
C5—C4A—C8A—N1	177.06 (17)	C18—C19—C20—C21	0.8 (3)
C4—C4A—C8A—N1	2.4 (3)	C19—C20—C21—C16	−1.9 (3)
C5—C4A—C8A—C8	1.1 (3)	C17—C16—C21—C20	1.2 (3)
C4—C4A—C8A—C8	−173.56 (17)	C15—C16—C21—C20	−178.09 (17)
C7—C8—C8A—N1	−176.50 (18)		

### 3-Benzyl-2-[(E)-2-(thiophen-2-yl)ethenyl]-2,3-dihydroquinazolin-4(1H)-one (II) Hydrogen-bond geometry (Å, º)

**Table:**

*D*—H···*A*	*D*—H	H···*A*	*D*···*A*	*D*—H···*A*
N1—H1···O1^i^	0.87 (3)	2.14 (3)	2.978 (2)	161 (2)

Symmetry code: (i) −*x*+1/2, *y*−1/2, *z*.
